# Characterization of Inflammatory Bowel Disease in the Elderly According to Age of Onset

**DOI:** 10.3390/jcm13247581

**Published:** 2024-12-13

**Authors:** Manuel Bracho González, Raúl Vicente Olmedo Martín, Ana Isabel Morales Bermúdez, Miguel Jiménez Pérez

**Affiliations:** UGC de Aparato Digestivo, Instituto de Investigación Biomédica de Málaga (IBIMA) Plataforma BIONAD, Hospital Regional Universitario de Málaga, 29010 Málaga, Spain; manuelbracho_4@hotmail.com (M.B.G.); raulolmedo1976@yahoo.es (R.V.O.M.); ana_m_bermudez@hotmail.com (A.I.M.B.)

**Keywords:** inflammatory bowel disease, elderly, IBD onset, Crohn’s disease, ulcerative colitis, biologic drugs, IBD-related surgeries

## Abstract

**Background/Objectives**: Elderly populations are under-represented in inflammatory bowel disease (IBD) clinical trials, with limited data on phenotype, treatment patterns, outcomes, and comorbidities. The main objective of this study was to evaluate, in an elderly cohort with IBD, demographic and disease characteristics, comorbidity, polypharmacy, and treatment patterns according to the development of IBD at or before old age. Secondarily, the same analysis was performed based on the type of IBD: ulcerative colitis (UC) or Crohn’s disease (CD). **Materials and Methods**: Observational, single-center, retrospective study including patients diagnosed with IBD and aged 65 years or older seen at the IBD office of the Regional University Hospital of Malaga between September and November 2022. Data were recorded on demographic, disease-related, and IBD treatment-related variables, comorbidities, and polypharmacy. A descriptive and analytical study was undertaken according to the age of IBD onset and type of IBD. **Results**: Of the patients included, 50.8% were male, 55.1% had CD, and 44.9% UC. IBD onset was before age 65 years in 69.5% and ≥65 years in 30.5%. Elderly with IBD who debuted <65 presented longer disease duration (19.67 ± 9.82 years) and required more IBD-related surgeries (37.8%); elderly with IBD who debuted ≥65 were older (77.69 ± 6.26 years), with no differences in the other variables. According to the type of IBD, elderly UC patients were older (74.55 ± 6.9 years), used more aminosalicylates (77.4%), and had higher rates of polypharmacy (90.6%). Elderly patients with CD had higher IBD activity (moderate/severe in 72.3%), used more biologic drugs (58.5%), and required more IBD-related surgeries (44.6%). **Conclusions**: Elderly patients who develop IBD before or after the age of 65 years are overall very similar in baseline and disease-related characteristics. Elderly with CD have higher IBD activity and require more biologic drugs and IBD-related surgeries. Elderly with UC are older and have higher rates of polypharmacy and aminosalicylate use.

## 1. Introduction

Inflammatory bowel disease (IBD) is a chronic disease with an uncertain course characterized by recurrent inflammation of the digestive tract. It has periods of worsening of symptoms (flares) interspersed with periods of quiescence or inactivity (remission). The main symptoms IBD produces include chronic diarrhea (with or without bleeding), abdominal pain, and weight loss [1,2,3].

The two main forms of IBD are ulcerative colitis (UC), which principally involves inflammation of the mucosal and submucosal layer of the colon (only involving muscularis mucosae in severe disease), and Crohn’s disease (CD), where involvement of the intestine or colon may be transmural and affect the whole digestive tract, from the mouth to the anus [4,5,6].

Although the etiopathogenesis of IBD is not certain, various studies suggest that the disease develops after an interaction between intestinal antigens and intestinal mucus, resulting in increased permeability and an altered barrier function, leading to an excessive immune response in certain patients with a genetic predisposition [7,8]. Other factors, such as diet, smoking, breastfeeding, or the consumption of antibiotics, NSAIDs, or contraceptives, also play an important role in disease development [9].

The diagnosis of IBD should be established from a combination of compatible clinical, biochemical, endoscopic, histologic, and radiologic findings, there being no gold standard for its diagnosis [10]. An increasing clinical suspicion of IBD, the use of non-invasive markers like fecal calprotectin, and a greater availability of diagnostic tests, such as colonoscopy, have resulted in an increase in the incidence and prevalence of the disease [11,12].

Two peaks have traditionally been described in the development of IBD, one between the ages of 20 and 40 years and the other between 60 and 70 years, with the latter group comprising 25–35% of all patients with IBD [13,14]. In Spain, one in every four patients with IBD is older than 65 years, thus representing an important proportion of all patients seen at the IBD office. This increase in the number of elderly patients with IBD is partly due to an increased incidence in this age group, as well as the aging of patients with a previous diagnosis of IBD [15,16].

The term older or elderly in IBD usually refers to patients who are older than 60 or 65 years of age, although in some publications it refers to patients older than 70 or even 75 years. Most studies in this age group do not differentiate between older patients with IBD who develop their disease in old age and those who were diagnosed when they were younger and have since aged [17,18]. However, the distinction between these two subgroups can be important, as differences may exist in epidemiology, phenotype, prognosis, and specific considerations concerning safety with the available treatments [19].

In general, older patients with IBD have an increased risk of severe and opportunistic infections, neoplasms, thromboembolic events, hospitalizations, and post-surgical morbidity and mortality, requiring greater consumption of medicines and ambulatory care [20,21,22,23]. However, this risk is related to the comorbidity associated not so much with chronological age but rather with cardiovascular problems, polypharmacy, and frailty—factors that must be considered when basing IBD treatment in the elderly. Special consideration should also be given to the clinical situation and prognostic factors, as in younger patients with IBD.

In addition, the elderly population with IBD has higher rates of malnutrition, osteoporosis, depression and anxiety disorders, and fatigue, as well as reduced physical activity, all leading to a significant reduction in their quality of life and functional status [24,25,26,27].

All these considerations hinder establishment of an optimum treatment strategy to manage IBD in elderly patients [28]. In general, we can state that the efficacy of the drugs available for the treatment of IBD seems similar in both young and elderly patients, though representation of older patients in clinical trials is scarce [14]. Those studies about the tendencies of treatment in an older population with IBD show a greater use of aminosalicylates and corticosteroids, with a lesser use of immunomodulatory and biological agents and small molecules, probably due to fear of adverse events [29,30,31].

Achieving clinical remission in IBD is especially important in this population, thereby reducing the risk of functional worsening and increasing the quality of life of elderly patients with IBD, who will have a more active and independent life [28]. All these considerations make it necessary to design new studies on the treatment of the older patient with IBD in real clinical practice, helping to define and optimize the therapeutic management of these patients.

Accordingly, we present this study of real clinical practice in a cohort of elderly patients with IBD. The principal objective was to evaluate in this cohort the demographic characteristics, together with data concerning the disease, comorbidity, polypharmacy, and treatment patterns depending on the age of onset of IBD, at old age or earlier. As a secondary aim, we undertook the same analysis in the patients according to their type of IBD, UC or CD.

## 2. Materials and Methods

### 2.1. Study Population and Design

This observational, single-center, retrospective study was undertaken at the Inflammatory Bowel Disease Unit of the Regional Hospital of Malaga. By consecutive sampling between September and November 2022, patients diagnosed with IBD and aged 65 years or older were included in the database. Patients were excluded if they did not have an established diagnosis of IBD or a routine follow-up in the IBD office. The diagnosis of IBD had to be recorded in the clinical history, previously defined according to a combination of compatible clinical, biochemical, endoscopic, histologic, and/or radiologic findings.

### 2.2. Variables

The following variables were recorded in the database.

#### 2.2.1. Principal Variable: Onset of IBD Before or After the Age of 65 Years

#### 2.2.2. Secondary Variables

–Demographic: sex, age at last visit, and smoking.

–Disease-related: type of IBD (CD or UC), localization in the case of UC (proctitis, left colitis, or extensive colitis), localization in the case of CD (ileum, colon, ileocolon, or upper intestine), phenotype in the case of CD (inflammatory, stenosing, penetrating, or any of the 3 with perianal involvement), years of duration of the IBD, disease activity at the last visit according to the evaluation of the IBD specialist based on clinical, biological, fecal, endoscopic, and/or radiologic data (disease in remission/mild or moderate/severe disease).

–Comorbidities. Graduated according to the Charlson comorbidity index, unadjusted for age, considering comorbidity to be mild (0–2 points), moderate (3–4 points), or severe (>4 points). The Charlson index associates patient comorbidity with long-term death, bearing in mind the presence or otherwise of the following 19 variables: myocardial infarction, diabetes mellitus without organ involvement, diabetes mellitus with organ involvement, congestive heart failure, hemiplegia, peripheral vascular disease, chronic kidney disease, cerebrovascular disease, solid tumor without metastasis, solid tumor with metastasis, leukemia, lymphoma, dementia, chronic obstructive pulmonary disease, connective tissue disease, mild liver disease, moderate or severe liver disease, peptic ulcer, and AIDS [32] (Table A1).

–Polypharmacy and total number of treatments. Based on the total number of chronic active treatments in the health service electronic and intra-hospital prescriptions, polypharmacy was considered to be the use of ≥5 treatments [33,34]. Drugs prescribed for an acute process were not counted in the total number of treatments.

–Treatment-related: active treatment for the IBD; chronic use of aminosalicylates; corticosteroids; topical treatment; immunomodulators; biologic/small molecule treatment, and if so, type (anti-TNF, ustekinumab, vedolizumab, or tofacitinib); IBD-related surgery.

### 2.3. Statistical Analysis

A descriptive study was undertaken of all the variables recorded, calculating the absolute and relative frequencies for the qualitative variables and the arithmetic mean and standard deviation (SD) for the quantitative variables. The 95% confidence interval of safety was estimated.

For the bivariate analysis, normality of the sample was assumed since both groups had at least 30 subjects. The Levene test was used to evaluate homogeneity. As the study design involved independent data with two study groups, the Student t test was used to compare the quantitative variables, the Mann–Whitney U test for the qualitative ordinal variables, and the Chi-square test for the qualitative nominal variables, after correction with Fisher’s exact test when necessary (if the expected frequency was less than 5). All the contrasts were bilateral and were considered to be significant if the *p* < 0.05. The data were collected, processed, and analyzed with IBM SPSS Statistics, version 29.0.1.0 (171).

### 2.4. Ethical and Legal Aspects

This study was conducted in accordance with the recommendations of the Declaration of Helsinki and was approved by the hospital’s Research Ethics Committee. The database was anonymized, and only the principal investigator had access to it.

## 3. Results

### 3.1. Descriptive Study

A total of 118 patients, aged 65 years or older, with an established diagnosis of IBD, were included. Table 1 shows the descriptive characteristics for the whole group.

### 3.2. Analytical Study According to the Age of IBD Onset

**Figure 1:**
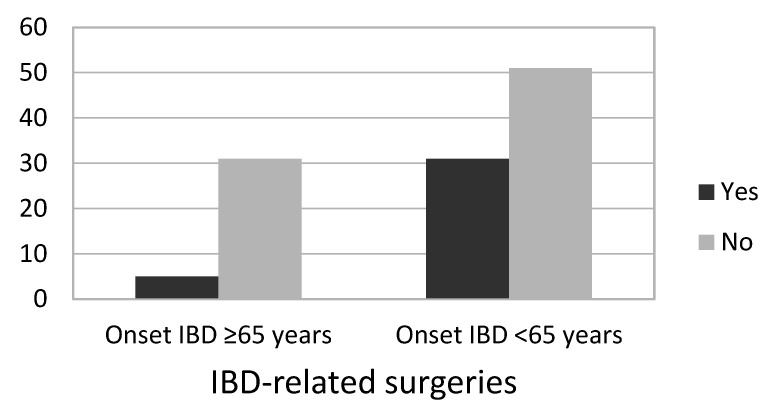
Need for IBD-related surgeries according to IBD diagnosis before or after the age of 65 years. IBD: inflammatory bowel disease.

Of the 118 study patients, 36 (30.5%) had received the diagnosis of IBD at age 65 or older (Group 1), and the remaining 82 (69.5%) had been diagnosed with IBD previously and had aged to at least 65 years (Group 2). The demographic characteristics, as well as data concerning disease, comorbidity, polypharmacy, and treatment patterns, were studied in our elderly patients with IBD according to whether they developed the disease in old age or before. Significant differences were found in the variables Need for IBD-related surgery (Figure 1) and Years of duration of the disease, in both cases greater in the group diagnosed with IBD < 65 years; and in the variable Age at last visit, in this case greater in the group diagnosed ≥65 years. No significant differences were seen in the other variables. The results of the full analytical study are shown in Table 2.

### 3.3. Analytical Study According to the Type of IBD

**Figure 2:**
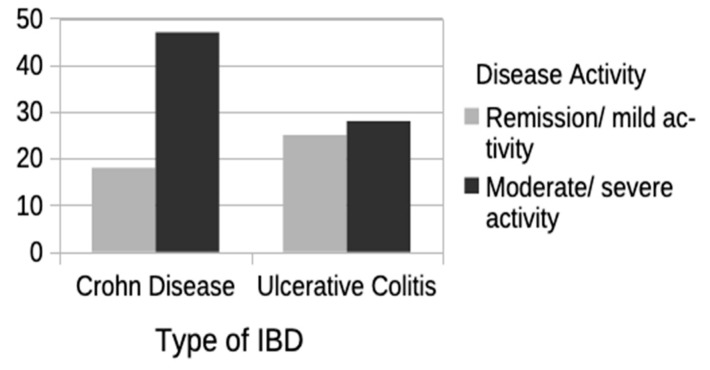
Disease activity according to type of IBD: Crohn’s disease or ulcerative colitis. IBD: inflammatory bowel disease.

**Figure 3:**
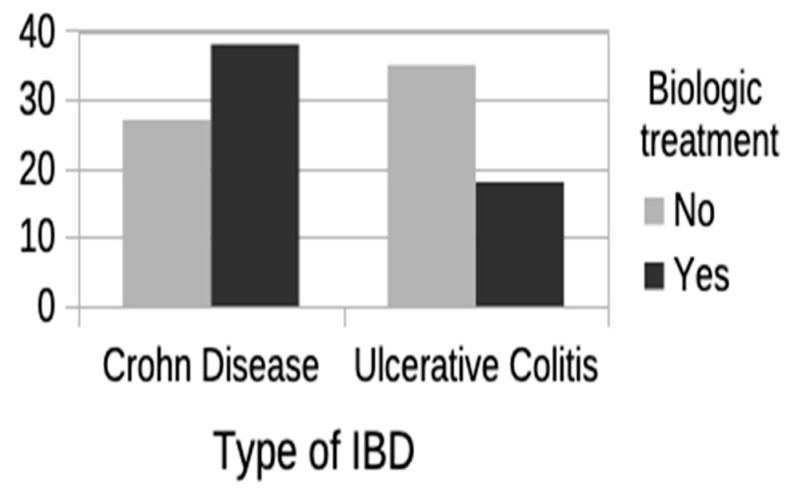
Use of biologic treatment according to type of IBD: Crohn’s disease or ulcerative colitis. IBD: inflammatory bowel disease.

Finally, a second analysis was performed, dividing the study population according to type of IBD: Crohn’s disease (Group 1) or ulcerative colitis (Group 2). The same characteristics were assessed in each group (demographic, disease-related, comorbidity, polypharmacy, and treatment patterns). Significant differences were found in the variables polypharmacy, use of aminosalicylates, and age at last visit, all greater in the group with UC, as well as in the variables disease activity (Figure 2), use of biologic treatment (Figure 3), and need for IBD-related surgery, this time greater in the group with CD. Significant differences were also noted in the variable sex, with more women in the group with CD and more men in the group with UC. No significant differences were seen for any of the other variables. Table 3 shows the results of this second analysis.

## 4. Discussion

Elderly patients are poorly represented in clinical trials on the management of IBD. In addition, most relevant studies have focused on the adverse effects in this population of the various therapies available, thus limiting treatment options for older adults [17]. Accordingly, over recent years, different papers and guidelines, as well as scientific societies, have published their position papers on IBD in the elderly, attempting to characterize this older population with IBD, identifying their differences in epidemiology, phenotype, prognosis, and specific considerations of efficacy and safety of the treatments available [14,17,18,28,29,35].

Concerning epidemiology in this population, our results show a predominance of men in the elder patients with UC and a predominance of women among those with CD. These results are in agreement with those of Taleban et al. [17] and Sturm et al. [14].

According to various studies [12,19,35], CD in the elderly is more often located in the colon, presenting an inflammatory pattern, whereas UC usually affects the left colon. On the other hand, our study found a predominance of ileal involvement in CD, with an inflammatory pattern, and a similar proportion between left and extensive colitis in UC.

Study of the treatment patterns in our sample showed that almost 80% of the elderly patients with UC were on chronic treatment with aminosalicylates, similar to the percentage reported by Taleban et al. (84%) [17]. However, this same study reported the use of aminosalicylates in 80% of the patients with CD [17], whereas only 18.5% of our patients with CD received this treatment. Chronic systemic steroids were used by 11.9% of our patients, much lower than the percentages reported by Juneja et al. [29] and Parian et al. [28] (35–40%). On the other hand, the use of immunomodulators (15.2%) and anti-TNF agents (12.7%) was greater in our patients than in those of Juneja et al., where immunomodulators were used by just 6% of the older patients with IBD and anti-TNF agents by 9% and 1% of older patients with CD and UC, respectively [29]. Overall, almost half the older population with IBD received biologic treatment, mainly ustekinumab. These differences in prescription patterns may be due to the fact that our sample was collected in a third-level hospital, where the patients have a more aggressive disease or are more difficult to manage.

Another aspect to consider in the treatment of IBD in the elderly is the frequency of polypharmacy, with the consequently greater risk of drug interactions and toxicity. Our study found a 76.3% rate of polypharmacy, with a mean of seven drugs, more frequently in older patients with UC. These results are similar to the prevalence of polypharmacy reported by Parian et al. in their study specifically evaluating this parameter in older patients with IBD (polypharmacy of 85%, with over 40% of their sample taking >10 drugs) [28]; and the mean number of medications reported by Juneja et al. in older patients with IBD (7 ± 3.5) [29].

Concerning the safety of the different treatment regimens, various studies have suggested that advanced age is a risk factor for the development of severe and opportunistic infections in IBD patients treated with corticoids, immunomodulators, and anti-TNF biologic agents [20,21,22,23,36]. A greater risk has also been reported for lymphomas [37] and skin cancer in older patients with IBD, particularly if they receive treatment with immunomodulators or anti-TNF drugs [38,39,40]. Nonetheless, this risk is related above all with cardiovascular comorbidity and frailty more than with chronologic age itself, as recently indicated by Kochar et al. in their study comprising over 11,000 patients with IBD [41]. Our study found a predominantly mild rate of comorbidity in our older patients with IBD (67.8%), with no obvious differences after stratifying the older patients with IBD according to age of onset before or after 65 years.

It is also worth noting that being over 50 years of age emerges as a predictor of physical inactivity [26,27]. This can be attributed to both IBD-related factors and age-related factors. Fatigue and increased prevalence of depression and anxiety disorders (even in periods of symptomatic remission of IBD) stand out [26]. Malnutrition is also more frequent in patients with IBD > 65 years than in younger ones [24]. These surrogate markers of frailty predispose the elderly population with IBD to complications such as sarcopenia and other exercise-related issues [24,25,27,42].

Distinguishing IBD in the elderly according to whether the onset is before or after 65 years may have important implications for the evolution of the disease and its prognosis, starting with the fact that IBD is usually more aggressive during the first year after its diagnosis and that the risk of infectious complications is greater in the initial months of treatment [43,44]. A retrospective study comparing older patients with IBD who developed the disease before or after 60 years of age showed that the overall disease-related complications were similar in both groups, though treatment-related complications were greater in the patients who developed IBD after the age of 60 years [45]. Our results are similar, showing that both groups (onset before or after 65 years) were comparable regarding the epidemiology, phenotype, disease activity, comorbidity, polypharmacy, and treatment patterns.

Finally, concerning the need for surgery related to IBD, older patients are reported to have greater rates of surgery, mainly soon after the diagnosis [46,47,48]. Our results, on the other hand, showed a greater need for surgery in the older patients who developed IBD before the age of 65 years, with a longer disease duration. In our sample, surgery was also more likely to be indicated in those patients with CD (44.6%), who also presented greater disease activity than the patients with UC.

This study, however, has certain limitations related to the fact that it was retrospective and single-center. Nevertheless, it has the strength of being carried out in real clinical practice and that it included a considerable number of patients. Of note, too, is the fact that comorbidity was assessed with the Charlson index, bearing in mind the clinical history of the patients, though in some cases this may not have been up to date. Disease activity was not measured formally with standardized indices but rather according to the overall assessment of the specialist in the IBD office. We were unable to evaluate treatment adherence by our older patients and, consequently, its importance in the presence or otherwise of polypharmacy. The fact that the study population was taken from a third-level hospital suggests that the sample represents patients with a more aggressive pattern of disease, with a greater use of advanced therapies in the treatment of their IBD (in particular biologic drugs).

Future perspectives on the subject of characterization of the elderly population with IBD could be developed with the help of multicenter studies, which would increase the statistical power of the study.

## 5. Conclusions

In our population of older patients who had IBD, no differences were found according to whether they developed the disease before or after the age of 65 years in demography, comorbidity, polypharmacy, treatment patterns, phenotype, or disease activity. However, the patients who developed the disease before the age of 65 years had a longer disease duration and required more surgical procedures. CD was predominant among the study population, more frequently affecting women, presenting a more active course with the need for more biologic treatments and more surgery than the patients with UC, which was more common among older men with greater rates of polypharmacy and greater use of aminosalicylates. There is a high overall use of advanced therapies in the treatment of IBD in the elder patient, particularly biologic drugs.

## Figures and Tables

**Table 1:** Characteristics of the study population. The results are presented as absolute and relative frequencies (for the qualitative variables) and arithmetic mean plus standard deviation (for the quantitative variables).

	N = 118
Sex	
Male	60 (50.8%)
Female	58 (49.2%)
Age at last visit (years)	72.93 (±6.21)
Smoking	
Yes	24 (20.3%)
No	94 (79.7%)
Type of IBD	
CD	65 (55.1%)
UC	53 (44.9%)
Localization in UC	53 (44.9%)
Proctitis (E1)	10 (8.5%)
Left colitis (E2)	22 (18.6%)
Extensive colitis (E3)	21 (17.8%)
Localization in CD	65 (55.1%)
Ileum (L1)	36 (30.5%)
Colon (L2)	12 (10.2%)
Ileum + colon (L3)	18 (15.3%)
Upper intestine (L4)	0 (0%)
Phenotype in CD	65 (55.1%)
Inflammatory (B1)	30 (25.4%)
Stenosing (B2)	12 (10.2%)
Penetrating (B3)	12 (10.2%)
B1, B2, or B3 with perianal involvement (*p*)	11 (9.3%)
Onset of IBD	
After age 65 years	36 (30.5%)
Before age 65 years	82 (69.5%)
Years of duration of the IBD	15.83 (±10.35)
Charlson comorbidity index	
Mild (0–2 points)	80 (67.8%)
Moderate (3–4 points)	29 (24.6%)
Severe (>4 points)	9 (7.6%)
IBD activity	
Remission/mild	43 (36.4%)
Moderate/severe	75 (63.6%)
Polypharmacy	
Yes	90 (76.3%)
No	28 (23.7%)
Total number of treatments	7.08 (±3.26)
Active treatment for IBD	
Yes	102 (89.8%)
No	12 (10.2%)
Chronic use of aminosalicylates	
Yes	53 (44.9%)
No	65 (55.1%)
Chronic use of systemic steroids	
Yes	14 (11.9%)
No	104 (88.1%)
Use of topical treatment	
Yes	24 (20.3%)
No	94 (79.7%)
Chronic use of immunomodulators	
Yes	18 (15.3%)
No	100 (84.7%)
Use of biologic or small molecule treatment	
Yes	56 (47.5%)
Anti-TNF	15 (12.7%)
Ustekinumab	29 (24.6)
Vedolizumab	12 (10.2%)
Tofacitinib	0 (0%)
No	62 (52.5%)
IBD-related surgery	
Yes	36 (30.5%)
No	82 (69.5%)

N: sample; IBD: inflammatory bowel disease; CD: Crohn’s disease; UC: ulcerative colitis; TNF: tumor necrosis factor.

**Table 2:** Stratified analysis based on age of IBD diagnosis. *p*-values < 0.05 are considered statistically significant and appear in bold. ^(a)^ Chi-square test; ^(b)^ Chi-square test with correction by Fisher’s exact test; ^(c)^ Mann–Whitney U test; ^(d)^ Student *t* test.

	Onset IBD ≥ 65 Years (*n* = 36)	Onset IBD < 65 Years (*n* = 82)	*p*-Value
Sex			
Male	15 (41.7%)	45 (54.9%)	0.19 ^(a)^
Female	21 (58.3%)	37 (45.1%)	
Smoking	7 (19.4%)	17 (20.7%)	0.87 ^(a)^
Type of IBD			
CD	18 (50%)	47 (57.3%)	0.46 ^(a)^
UC	18 (50%)	35 (42.7%)	
Localization in UC			
E1	4 (22.2%)	6 (17.1%)	0.9 ^(a)^
E2	7 (38.9%)	15 (42.9%)	
E3	7 (38.9%)	14 (40%)	
Localization in CD			
L1	12 (66.7%)	24 (50%)	0.42 ^(a)^
L2	3 (16.7%)	9 (18.8%)	
L3	3 (16.7%)	15 (31.3%)	
Phenotype in CD			
B1	11 (61.1%)	19 (40.4%)	0.24 ^(a)^
B2	4 (22.2%)	8 (17%)	
B3	1 (5.6%)	11 (23.4%)	
B1, B2, or B3 + *p*	2 (11.1%)	9 (19.1%)	
Polypharmacy	29 (80.6%)	61 (74.4%)	0.47 ^(a)^
Active treatment for IBD	34 (94.4%)	72 (87.8%)	0.34 ^(b)^
Chronic use of aminosalicylates	21 (58.3%)	32 (39%)	0.052 ^(a)^
Chronic use of systemic steroids	5 (13.9%)	9 (11%)	0.76 ^(b)^
Topical treatment	11 (30.6%)	13 (15.9%)	0.07 ^(a)^
Immunomodulators	3 (8.3%)	15 (18.3%)	0.17 ^(a)^
Biologic treatment	14 (38.9%)	42 (51.2%)	0.22 ^(a)^
Type of biologic agent			
Anti-TNF	3 (21.4%)	12 (28.6%)	0.72 ^(a)^
Ustekinumab	7 (50%)	22 (52.4%)	
Vedolizumab	4 (28.6%)	8 (19%)	
IBD-related surgery	5 (13.9%)	31 (37.8%)	**0.009** ^(a)^
IBD activity			
Remission/mild	13 (36.1%)	30 (36.6%)	0.96 ^(c)^
Moderate/severe	23 (63.9%)	52 (63.4%)	
Charlson comorbidity index			
Mild (0–2)	25 (69.4%)	55 (67.1%)	0.74 ^(c)^
Moderate (3–4)	9 (25%)	20 (24.4%)	
Severe (>4)	2 (5.6%)	7 (8.5%)	
Age at last visit (years)	77.69 (±6.26)	70.84 (±4.93)	**<0.001** ^(d)^
Years of duration of the IBD	7.08 (±4.64)	19.67 (±9.82)	**<0.001** ^(d)^
Total number of treatments	7.47 (±3.11)	6.91 (±3.32)	0.39 ^(d)^

N: sample; IBD: inflammatory bowel disease; CD: Crohn’s disease; UC: ulcerative colitis; E1: proctitis; E2: left colitis; E3: extensive colitis; L1: ileum; L2: colon; L3: ileum + colon; L4: upper intestine; B1: inflammatory; B2: stenosing; B3: penetrating; *p*: perianal involvement; TNF: tumor necrosis factor. The results are presented as absolute and relative frequencies (for the qualitative variables) and arithmetic mean plus standard deviation (for the quantitative variables).

**Table 3:** Stratified analysis according to type of IBD. *p*-values < 0.05 are considered statistically significant and appear in bold. ^(a)^ Chi-square test; ^(b)^ Mann–Whitney U test; ^(c)^ Student *t* test.

	CD (*n* = 65)	UC (*n* = 53)	*p*-Value
Sex			
Male	27 (41.5%)	33 (62.3%)	**0.025** ^(a)^
Female	38 (58.5%)	20 (37.7%)	
Smoking	16 (24.6%)	8 (15.1%)	0.2 ^(a)^
Polypharmacy	42 (64.6%)	48 (90.6%)	**<0.001** ^(a)^
Active IBD treatment	56 (86.2%)	50 (94.3%)	0.14 ^(a)^
Chronic aminosalicylates	12 (18.5%)	41 (77.4%)	**<0.001** ^(a)^
Chronic systemic steroids	9 (13.8%)	5 (9.4%)	0.46 ^(a)^
Immunomodulators	13 (20%)	5 (9.4%)	0.11 ^(a)^
Biologic treatment	38 (58.5%)	18 (34%)	**0.008** ^(a)^
Type of biologic used			
Anti-TNF	12 (31.6%)	3 (16.7%)	0.48 ^(a)^
Ustekinumab	18 (47.4%)	11 (61.1%)	
Vedolizumab	8 (21%)	4 (22.2%)	
IBD-related surgery	29 (44.6%)	7 (13.2%)	**<0.001** ^(a)^
IBD activity			
Remission/mild	18 (27.7%)	25 (47.2%)	**0.029** ^(b)^
Moderate/severe	47 (72.3%)	28 (52.8%)	
Charlson comorbidity index			
Mild (0–2)	45 (69.2%)	35 (66%)	0.84 ^(b)^
Moderate (3–4)	14 (21.5%)	15 (28.3%)	
Severe (>4)	6 (9.2%)	3 (5.7%)	
Age at last visit (years)	71.62 (±5.29)	74.55 (±6.9)	**0.013** ^(c)^
Years of duration of the IBD	15.98 (±11.05)	15.64 (±9.52)	0.86 ^(c)^
Total number of treatments	6.63 (±3.71)	7.64 (±2.51)	0.082 ^(c)^

N: sample; IBD: inflammatory bowel disease; CD: Crohn’s disease; UC: ulcerative colitis; TNF: tumor necrosis factor. The results are presented as absolute and relative frequencies (for the qualitative variables) and arithmetic mean plus standard deviation (for the quantitative variables).

## Data Availability

Date are contained within the article.

## Appendix A

**Table A1:** Charlson comorbidity index: components and score.

Comorbidity	Score
Myocardial infarction	1
Congestive heart failure	1
Peripheral vascular disease	1
Cerebrovascular disease	1
Dementia	1
Chronic obstructive pulmonary disease	1
Connective tissue disease	1
Peptic ulcer	1
Mild liver disease	1
Diabetes without organ involvement	1
Hemiplegia	2
Moderate or severe renal disease	2
Diabetes with end-organ damage	2
Non-metastatic solid tumor	2
Leukemia	2
Lymphoma	2
Moderate or severe liver disease	3
Metastasis	6
AIDS	6
